# Healthcare knowledge of relationship between time series electrocardiogram and cigarette smoking using clinical records

**DOI:** 10.1186/s12911-020-1107-2

**Published:** 2020-07-09

**Authors:** Kuo-Kun Tseng, Jiaqian Li, Yih-Jing Tang, Ching Wen Yang, Fang-Ying Lin

**Affiliations:** 1 School of Computer Science, Harbin Institute of Technology (Shenzhen), Shenzhen, China; 2 Department of Family Medicine, Center for Geriatrics and Gerontology, Taichung Veterans General Hospital, Taichung, Taiwan; 3 Computer & Communication Center, Taichung Veterans General Hospital, Taichung, Taiwan; 4 School of Economics and Management, Harbin Institute of Technology (Shenzhen), Shenzhen, China

**Keywords:** Electrocardiogram, Smoking, Diagnostic system, Neural networks

## Abstract

**Background:**

In the few studies of clinical experience available, cigarette smoking may be associated with ischemic heart disease and acute coronary events, which can be reflected in the electrocardiogram (ECG). However, there is no formal proof of a significant relationship between cigarette smoking and electrocardiogram results. In this study, we therefore investigate and prove the relationship between electrocardiogram and smoking using unsupervised neural network techniques.

**Methods:**

In this research, a combination of two techniques of pattern recognition; feature extraction and clustering neural networks, is specifically investigated during the diagnostic classification of cigarette smoking based on different electrocardiogram feature extraction methods, such as the reduced binary pattern (RBP) and Wavelet features. In this diagnostic system, several neural network models have been obtained from the different training subsets by clustering analysis. Unsupervised neural network of clustering cigarette smoking was then implemented based on the self-organizing map (SOM) with the best performance.

**Results:**

Two ECG datasets were investigated and analysed in this prospective study. One is the public PTB diagnostic ECG databset with 290 samples (age 17–87, mean 57.2; 209 men and 81 women; 73 smoking and 133 non-smoking). The other ECG database is from Taichung Veterans General Hospital (TVGH) and includes 480 samples (240 smoking, and 240 non-smoking). The diagnostic accuracy regarding smoking and non-smoking in the PTB dataset reaches 80.58% based on the RBP feature, and 75.63% in the second dataset based on Wavelet feature.

**Conclusions:**

The electrocardiogram diagnostic system performs satisfactorily in the cigarette smoking habit analysis task, and demonstrates that cigarette smoking is significantly associated with the electrocardiogram.

## Background

Electrocardiography has a basic role in cardiology as it involves effective, simple, non-invasive and low-cost procedures for the diagnosis of cardiovascular disorders. Such disorders have a high epidemiologic incidence, and are of particular significance due to their impact on patient life and social costs. The ECG signal is a one-dimensional data set representing the time of electrical change of the voltage variation, which is detected on the skin. ECG technology has existed for over a century since its use for the first time in the clinic in 1903. In this century, the rapidly developing ECG technology has made great contributions to human life and health, as well as to developments in biological and clinical studies, and has been an indispensable routine examination technology in the clinic.

The solid relationship between the ECG and cardiovascular diseases is well known, and such diseases can be automatically classified and recognised based on the different ECG features. However, according to some research findings, the ECG is also connected with other diseases and habits such as diabetes mellitus and smoking. Cigarette smoking is a major preventable risk factor for coronary artery disease and sudden cardiac death [1], which is directly reflected in the ECG. Therefore, cigarette smoking is also significantly associated with ECG.

Most people might be aware that smoking causes lung cancer. The fact that cardiovascular disease also claims the lives of many smokers is less known. There are a number of cardiovascular diseases that are associated with smoking [2]. These include heart disease, stroke and peripheral vascular disease. Whenever a person smokes a cigarette, the chemicals in the smoke, particularly nicotine and carbon monoxide, damage the cardiovascular system. Nicotine causes both immediate and long-term increases in blood pressure, heart rate, cardiac output and coronary blood flow. Carbon monoxide binds to haemoglobin, which normally carries oxygen from the lungs via the bloodstream, and therefore reduces the amount of oxygen reaching body tissues. Smoking also promotes stickiness of both blood vessels and blood cells, allowing cholesterol and other dangerous fatty materials to build up inside blood vessels. This is termed atherosclerosis and can in turn can lead to raised blood pressure and clot formation.

Cigarette smoking affects the cardiovascular system [3], and cardiovascular diseases are tightly associated with the ECG. Cigarette smoking will therefore indirectly influence the features of the ECG, and this has been verified by the studies outlined below.

Cigarette smoking is a well-established risk factor for coronary artery disease (CAD) and its complications such as acute myocardial infarction and sudden death [1, 4]. More recently, several studies have investigated the effect of cigarette smoking on left and right ventricular function [5–7].

In a previous study [8], it was found that the short-term action of smoking consists of a brief fall in arterial pressure and heart rate occurring over eight to 10 heart beats immediately following the first inhalation of tobacco smoke. This is followed by a rebound rise in arterial pressure to a level greater than the pre-smoking level, most likely a vagal effect. Cigarette smoking has been shown to cause angina pectoris in one individual, and the records showed ST-segment depression in the ECG before the subjective appreciation of pain.

Cigarette smoking may also influence the prevalence of ECG Q-waves [9]. A higher prevalence of hypertension and of ECG Q-waves was observed in male smokers. Additionally, it was found that a group comprised of no-alcohol-consuming smokers had a significantly higher prevalence of hypertension and ECG Q-waves.

The aim of this paper was therefore to formally investigate the correlation between cigarette smoking and ECG results. This was based the electrocardiogram diagnostic system [10] combining the two techniques of clustering analysis and neural networking with different ECG features, such as reduced binary pattern (RBP) and Wavelet features. The experiments were performed in two ECG datasets: the PTB diagnostic ECG dataset [11] and an ECG dataset from TVGH.

## Methods

### Diagnostic system

**Fig. 1:**
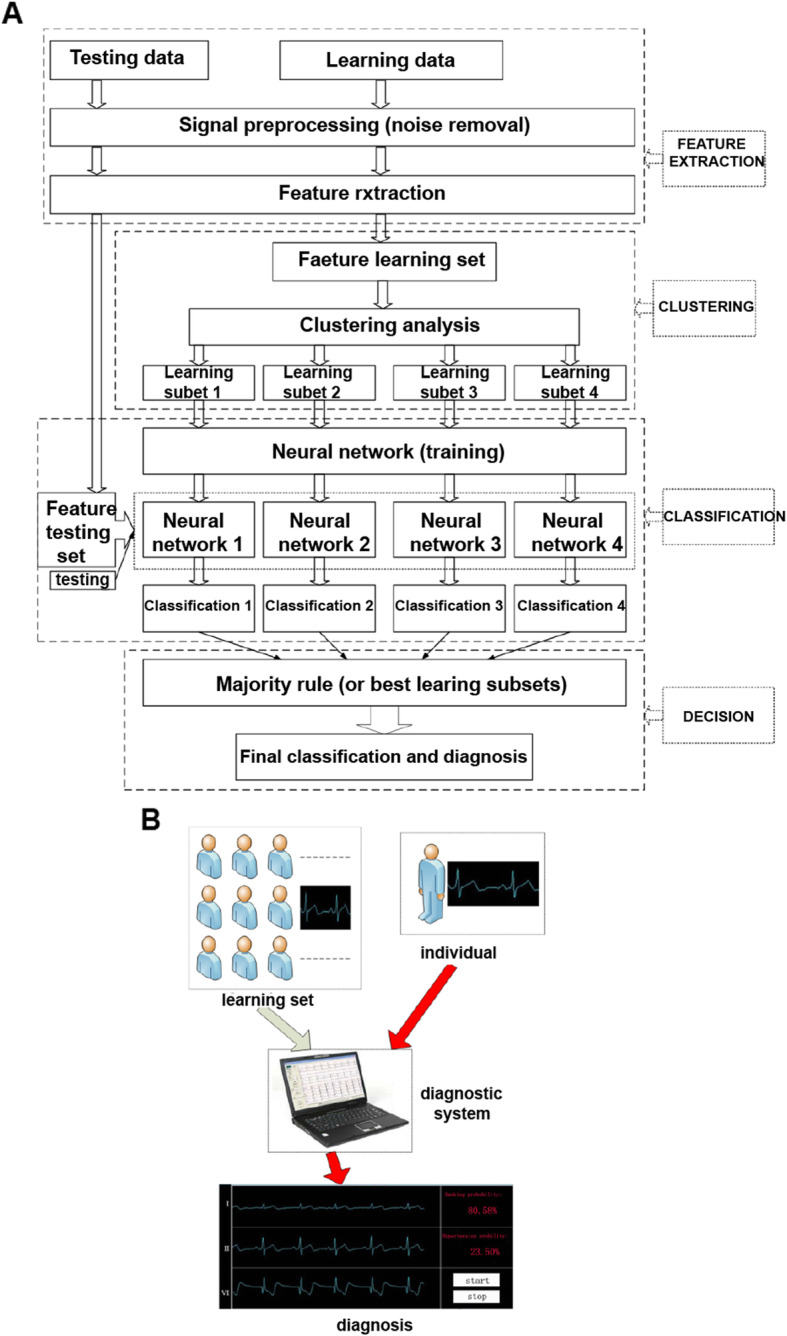
Electrocardiogram diagnostic system; (**a**) the structure and process of diagnostic system; (**b**) the application of diagnostic system

As is shown in Fig. 1, the diagnostic system consisted of four main chronological components: feature extraction, clustering, classification and decision. Since the system is provided with a set of ECG features such as RBP features with different scales as input, we first needed to pre-process the ECG signal to remove noise. The ECG features were subsequently extracted at the first stage of diagnostic process. During the ECG recording, the signal may be corrupted by low-frequency or high-frequency noise which alters the waveform of the ECG trace from its original structure. To eliminate this, the most common types of noise must be considered [12].

Extraneous noise in the ECG trace may be caused by a variety of noise sources including perspiration, respiration, body movements and poor electrode contact. Electrode motion artifacts manifest as large-amplitude waveforms primarily caused by stretching, which alters the impedance of the skin around the electrode. Power line interference is high-frequency noise caused by interference from nearby devices resulting from improper grounding of the ECG equipment. Electromyography (EMG) noise [13] is caused by the electrical activity of skeletal muscles during periods of contraction, or resulting from a sudden body movement. Although the frequency component of EMG overlaps considerably with that of the QRS complex, it also extends into higher frequencies. Commonly used filtering methods include the traditional FIR (Finite Impulse Response) filter method [14, 15], wavelet transform method [16, 17] and the EEMD (Ensemble Empirical Mode Decomposition) method [18].

The main purpose of the clustering step is to apply the best learning set for the neural network instead of the time-consuming, complex and inefficient method of handpicking the better learning set from the large dataset. Compared with the K-means clustering, the neural network clustering is more applicable to large data sets. The computational complexity scales linearly with the number of data samples, does not require huge amounts of memory (basically just the prototype vectors and the current training vector) and can be implemented in a neural, on-line learning manner as well as a parallelized [19] manner.

Based on the clustering step, and regarding the subsets of clustering as the learning subset of the neural network, several different neural network nets were trained. After the testing set was input into the nets above, the respective classifications corresponding to different nets were then obtained. This was the classification step.

There are many methods utilised at the stage of decision. Here, two simple and useful rules to combine all classifications from different neural network nets are introduced. One is the majority rule, and another is finding the best learning subset. The majority rule means that the minority is subordinate to the majority, and finding the best training subset requires that we choose the classification of the net with the best performance as the final classification and diagnosis.

### Clustering analysis module

This clustering analysis module is adapted from Self-Organising Map (SOM), it is proposed by Teuvo Kohonen [20, 21] to provide a data visualisation technique which helps to understand high dimensional data by reducing the dimensions of the data to a map. It is one of the neural network clustering methods. The SOM also represents a clustering concept by grouping similar data together. Therefore, it can be said that SOM reduces data dimensions and displays similarities among data.

The SOM consists of a regular, usually two-dimensional (2-D) grid of map units. Each unit is represented by a prototype vector *m*_*i*_ = [*m*_*i*1_, ⋯, *m*_*id*_], where *d* is the input vector dimension. The units are connected to the adjacent ones by a neighborhood relation. The number of map units, which typically varies from a few dozen up to several thousand, determines the accuracy and generalisation capability of the SOM. During training, the SOM forms an elastic net that folds onto the ‘cloud’ formed by the input data. Data points lying near each other in the input space are mapped onto nearby map units. Thus, the SOM can be interpreted as a topology that preserves mapping from input space onto the 2-D grid of map units.

The SOM is trained iteratively. At each training step, a sample vector *x* is randomly chosen from the input data set. Distances between *x* and all the prototype vectors are computed. The best-matching unit (BMU) is the map unit with prototype closest to x. Next, the prototype vectors are updated. The BMU and its topological neighbors are moved closer to the input vector in the input space according to the update rules. In the case of a discrete data set and fixed neighborhood kernel, there is an error function in the update process of the SOM.

The simplicity of the SOM is particularly attractive, with those SOMs close together with grey connecting them considered similar and those with a large black connecting region regarded as different. Unlike Multidimensional Scaling or N-land, the most effective method of use can be quickly picked up by the user [22].

### Neural network module

Neural network module is applied with backpropagation neural network [23] which provides a robust approach to approximating real-valued, discrete-valued, and vector-valued target functions. For certain types of problems, such as learning to interpret complex real-world sensor data, artificial neural networks are among the most effective learning methods currently known. For example, the backpropagation algorithm has proven surprisingly successful in many practical problems such as learning to recognise handwritten characters and spoken words.

The backpropagation algorithm [24] learns the weights for a multilayer network, given a network with a fixed set of units and interconnections. The learning problem faced by backpropagation is to search a large hypothesis space defined by all possible weight values for all the units in the network. It employs gradient descent to attempt to minimise the squared error between the network output values and the target values for these outputs in order to find the best hypothesis in the hypothesis space mentioned above.

One major difference in the case of multilayer networks is that the error surface can have multiple local minima, in contrast to the single-minimum parabolic error surface. Unfortunately, this means that gradient descent is guaranteed only to converge toward some local minimum, and not necessarily the global minimum error. Despite this obstacle, backpropagation has been found to produce excellent results in many real-world applications.

### Feature extraction module

Most ECG-based human disease analysis methods rely on feature extraction derived from ECG signals. The features are usually extracted according to three models: transform-based, waveform-based and statistical-based.

The transform-based algorithms consist of wavelet transforms [25, 26] and frequency domain transforms [12], including Fourier transform [27] or [DCT [28]]. Since the wavelet transform contains information in the time and frequency domain, it is more popular than the frequency domain transform.

Waveform-based algorithms extract different time domain characteristics (distance, height and area) from fiducial points inside the ECG waveform. These waveform descriptors are used to match or classify ECG signals in the disease analysis process. These algorithms usually have good accuracy in recognising regular ECG signals but show opposite results for irregular data.

Some researchers have combined a precision-matched result with a waveform neural network in the signal pre-processing stage [29]. This model extracted seven features from the ECG signals based on their amplitude and the interval to be analysed by the decision-based neural network. The computational complexity depends heavily on the forms of those time-domain ECG signals and the level of difficulty of the matching process carried out by the neural network. Nineteen characteristics are extracted from the time interval, amplitude and angle of deflection and then studied [30], with the classification being examined using Euclidian distances and an adaptive threshold. The eigenvectors used in feature matching take time but are necessary for all band waves in the ECG signals.

An ECG signal can be described as a non-stationary time series that presents some irregularities in the waveform. Unlike waveform-based algorithms, transform-based algorithms analyse the non-stationary information based on the presentation of the signal in the frequency domain. Not only is this process slow, but it is also difficult to extract good features for the purpose of classification.

Statistical-based algorithms usually depend on statistical evaluations (count, mean and variance) of human identification. They are usually less time-consuming but definitely require a well-designed statistical model to ensure high-quality accuracy. A method based on rank order statistics has been previously used to analyse the human heart beat [31].

1. Reduced Binary Pattern (RBP)

The concept of our proposed algorithm, RBP, for ECG disease is related to the work of Yang [31] and Kuma [32], although we expand it to a different field of application. The basic RBP algorithm [33] in our design can be roughly divided into two necessary steps that will be illustrated as follows.

**Fig. 2:**
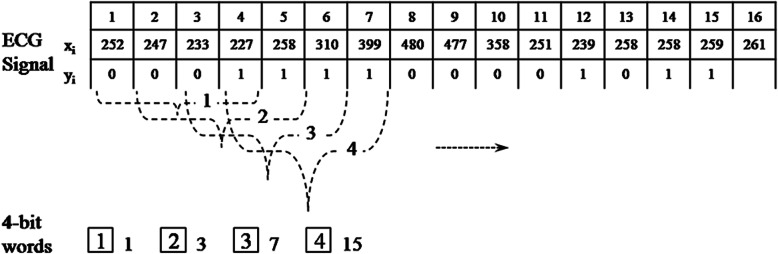
Reduced binary pattern conversion

The first step is a reduced binary pattern conversion. All ECG signals are non-stationary. Consider an ECG signal as a vector; each pair of consecutive input signals is compared and the data are categorised as either a decrease or an increase. A preliminary reduced function then maps these two cases to zero or one respectively, and if the former data set is larger than the latter data set, then the function value is one, otherwise, it is zero. This procedure converts the ECG signal to a binary sequence. Every m-bit in the binary sequence is grouped to construct a reduced binary sequence of length m, referred to as an m-bit word, and all such words are then collected to form a reduced binary pattern vector. Subsequently, we convert the m-bit word in the reduced binary pattern into its decimal format expansion. An example of the reduced binary pattern conversion for *m* = 4 is depicted in Fig. 2.

The next step, counting and ranking, is to count the occurrence of all decimal values and sort them in order of descending frequency. The frequency of each decimal value occurring in the decimal sequence is then calculated, and the relative frequency array is considered as the reduced binary pattern feature of the ECG signal. This will be used as the input for the clustering analysis and neural networks.

**Fig. 3:**
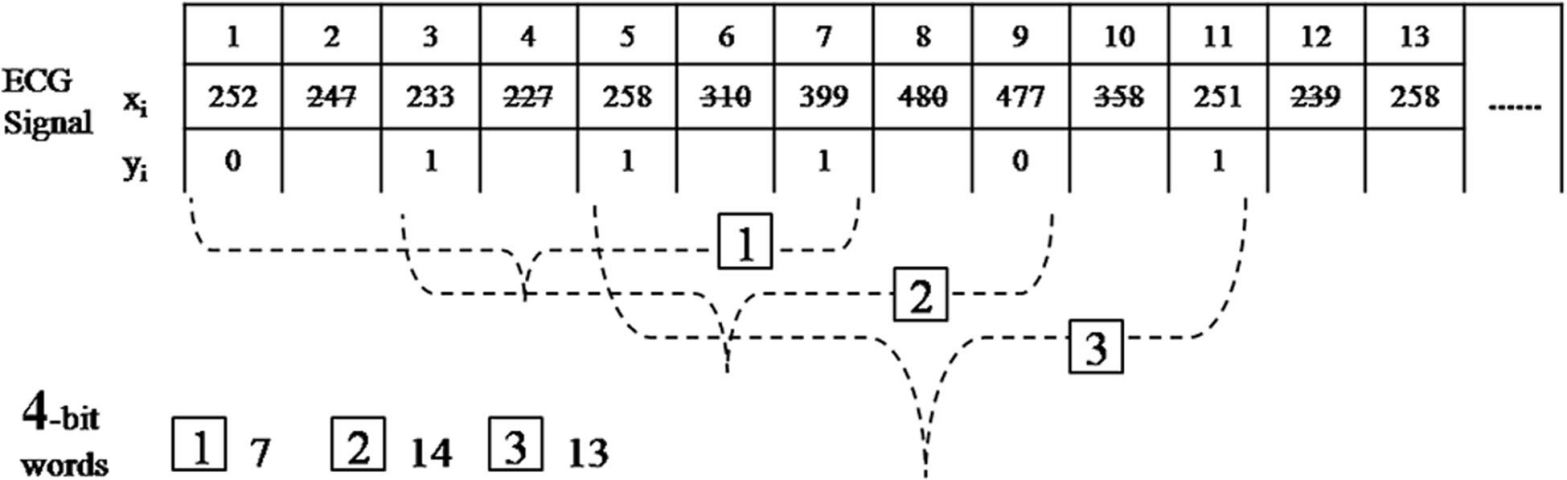
The modified reduced binary pattern conversion

Sometimes, since the basic RBP algorithms cannot achieve a positive performance in the electrocardiogram diagnostic system, we need add some variables to modify the algorithm, to make the algorithm more flexible, robust and applicative. For example, we consider a new scaling factor, which is an increment in the length of the interval. All steps in this advanced algorithm are similar to those performed in the basic design mentioned above, with the exception that the binary sequence will be obtained from the comparison of two data points with a specified distance instead of the two adjacent and consecutive values. Figure 3 represents the process of the modified RBP conversion of m = 4 and a scaling factor of 2.

2) Wavelet feature

**Fig. 4:**
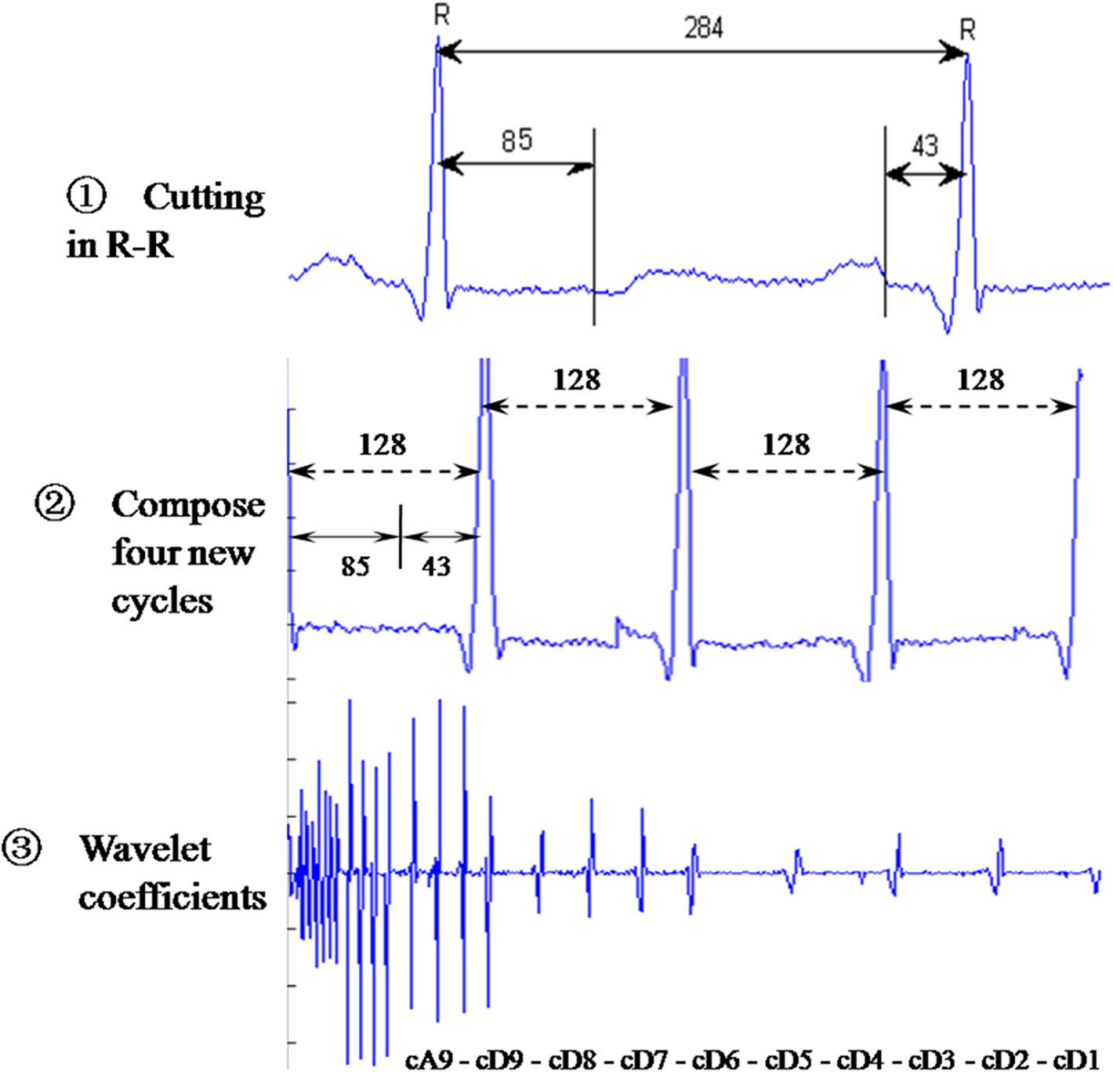
Process of extracting wavelet-based features

In comparison, the processes of the wavelet-based algorithm [25] include the following; each R-R cardiac cycle is obtained through R-R detection, an interpolation is performed on the R-R interval so that each R-R cardiac cycle holds 284 data points, every R-R cycle is cut into three parts, each containing 85, 156 and 43 points, the first 85 and the last 43 points in each R-R cycle are assembled to form a 128-point segment and every four segments are grouped and an n-level discrete wavelet transform (DWT) is performed to obtain the corresponding wavelet coefficients. Four of the computed wavelet coefficients are gathered as a wavelet vector. Here we use the coefficients vector of discrete wavelet transform as the ECG feature. An example with *n* = 9 is illustrated in Fig. 4. However, the R-R interval occasionally holds different a number of data points, which may be significantly more than 284. In this case we need choose the suitable length of the segments extracted from the ECG raw data. For example, 128, 256 or 512 data points can be obtained from every R-R cycle. However, the length of final combination segments cannot be too long, as this may make the clustering analysis and neural networks inefficient and time-consuming.

The position of the R points should be determined before extracting segments from ECG data. There are a large number of methods [34] available to detect the position of the R wave, such as algorithms based on amplitude and first derivative, algorithms based on first and second derivative, algorithms based on digital filters, algorithms based on wavelet transform and algorithms based on morphology and difference. Each method has its own advantages and disadvantages, for example, some algorithms are sensitive to ECG noise, while some algorithms have low robustness.

Here we have chosen a real-time QRS detection algorithm [35] for detection of the QRS complexes of ECG signals. It reliably recognises QRS complexes based upon the digital analysis of slope, amplitude and width. A special digital band pass filter reduces false detections caused by the various types of interference present in ECG signals. This filtering permits the use of low thresholds, thereby increasing detection sensitivity. The algorithm automatically adjusts thresholds and parameters periodically to adapt to such ECG changes as QRS morphology and heart rate.

## Results

### ECG datasets

As mentioned above, two ECG datasets were used in this experiment. One is the PTB diagnostic ECG dataset 11 and another is the ECG dataset from Taichung Veterans General Hospital (TVGH). The PTB dataset contains 549 records from 290 subjects. Subjects were aged between 17 and 87 (mean 57.2), with 209 men (mean age 55.5), and 81 women (mean age 61.6). Ages were not recorded for 1 female and 14 male subjects. Each subject is represented by one to five records. There are no subjects numbered 124, 132, 134 or 161. Each record includes 15 simultaneously measured signals: the conventional 12 leads (i, ii, iii, avr, avl, avf, v1, v2, v3, v4, v5 and v6) together with the 3 Frank lead ECGs (vx, vy and vz). Each signal is digitised at 1000 samples per second, with 16-bit resolution over a range of ±16.384 mV. On special request to the contributors of the dataset, recordings may be available at sampling rates up to 10 KHz. In this experiment, we only use the i lead as the raw source.

Within the header (.hea) file of most of these ECG records is a detailed clinical summary including age, gender, diagnosis, and where applicable, data on medical history, medication and interventions, coronary artery pathology, echocardiography and haemodynamics. Meanwhile, there are 73 smoking records and 133 non-smoking records for the experiments.

**Fig. 5:**
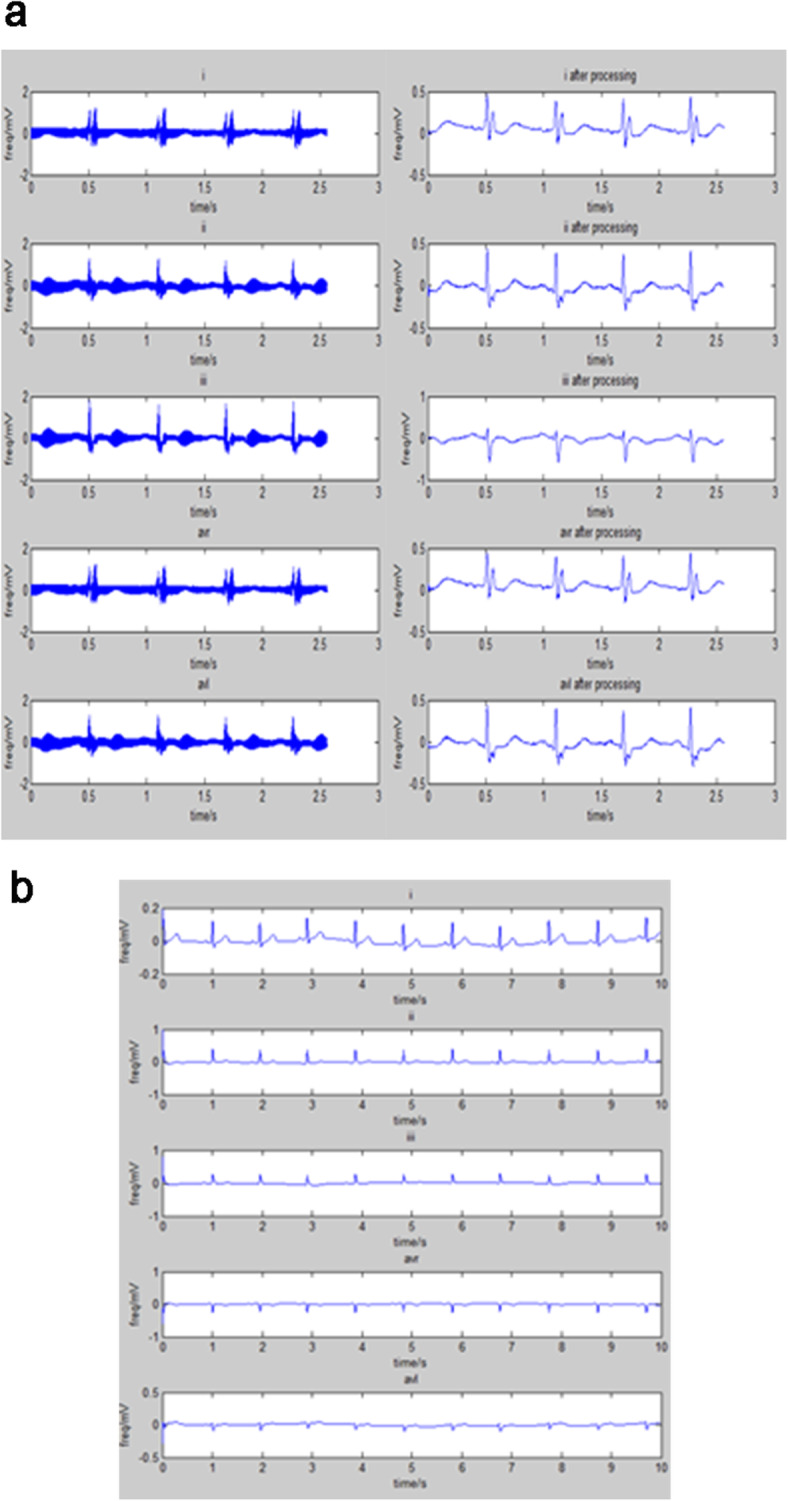
(**a**) The signal leads i, ii, iii, avr and avl in the PTB database. (**b**) The signal leads i, ii, iii, avr and avl in the second ECG database

In the ECG dataset from the TVGH, there are 480 records including 240 smoking records and 240 non-smoking records. Each record includes the conventional 12 leads (i, ii, iii, avr, avl, avf, v1, v2, v3, v4, v5 and v6), and each signal is digitised at 500 samples per second. The number of samples in each lead is 5500. Some information including age, sex, date of birth, weight, height, past medical history, personal medical history and family medical history are described in the case files. Since the raw data in the PTB diagnostic ECG dataset is severely affected by noise, the raw data requires pre-processing to eliminate the noise. The quality of signal in the ECG dataset from the TVGH is better that in the PTB dataset, as is shown in Fig. 5.

### Classification accuracy

As mentioned above, the two datasets differ in data quality and processing methods, and therefore may require different ECG features to improve the performance of the electrocardiogram diagnostic system. Throughout the experiments, we found that the RBP feature is significantly more suitable for the PTB dataset, that is, when we use the RBP feature with different scales to first clustering and subsequently classify, the accuracy of the electrocardiogram diagnostic system is higher than that with the wavelet feature. Furthermore, if we use the rule of finding the best learning subsets to make classification decisions, the result is slightly better than that of majority rule. In cases where we first choose the best learning subsets from the results of SOM clustering and then train the neural network which is provided with the best learning subsets as input, the accuracy is shown in Table 1. In Table 1, *m* denotes the number of bits in a word for the reduced binary pattern and *α* denotes the scaling factor of the interval for the reduced binary sequence. The best performance of this decision mechanism reached 80.58%.

**Table 1:** Best learning subset: accuracy with different *m* and α in the PTB database

m\α	1	2	4	8	16	32
4	68.45%	66.99%	75.73%	72.33%	67.96%	67.96%
5	65.53%	67.96%	68.45%	70.39%	67.96%	67.96%
6	66.99%	72.33%	71.36%	69.90%	68.93%	65.05%
7	68.93%	69.42%	75.24%	73.30%	68.45%	68.45%
8	69.42%	67.96%	76.70%	80.58%	67.48%	67.96%
9	66.99%	66.50%	73.79%	78.16%	71.84%	70.39%
10	68.93%	78.64%	80.10%	77.18%	69.90%	68.45%
11	72.33%	77.67%	75.24%	66.02%	68.93%	70.39%

If we firstly train different neural networks based on the subsets from the SOM clustering, and subsequently combine the outputs of different neural networks by majority rule, the performance, shown in Table 2, is slightly worse than in Table 1. The highest accuracy is just 74.76% when *m* = 11 and *α* = 2, which is lower than that in the decision mechanism finding the best learning subsets.

**Table 2:** Majority rule: accuracy with different m and α in the PTB database

m\α	1	2	4	8	16	32
4	67.96%	67.48%	70.39%	71.84%	70.39%	66.99%
5	66.99%	68.45%	69.42%	69.90%	67.96%	68.45%
6	66.50%	69.90%	69.90%	67.96%	69.42%	65.53%
7	67.48%	67.96%	72.82%	69.90%	67.48%	66.50%
8	69.42%	70.87%	71.84%	73.79%	69.42%	67.48%
9	66.50%	67.48%	68.93%	71.36%	66.50%	67.96%
10	70.87%	72.82%	69.90%	72.33%	71.36%	66.99%
11	66.50%	74.76%	74.27%	65.05%	66.99%	67.96%

However, for the second ECG dataset, accuracy with RBP features is much worse than in the first dataset. After the experiments, it was found that the performance of the diagnostic system with the Wavelet feature is positive. Therefore, the Wavelet feature was utilised for the diagnostic system instead of the RBP feature. Because each signal is digitised at 500 samples per second and the signal cycle contains more than 500 samples, 256 data points can be obtained from every R-R cycle. The first 169 and the last 85 points in each R-R cycle are assembled to form a 256-point segment; every four segments are grouped and an *n* level discrete wavelet transform (DWT) is performed to obtain the corresponding wavelet coefficients as the Wavelet feature. Here, *n* is between 1 and 9 to extract different features, in order to increase the accuracy of the diagnostic system.

Since the number of samples in the second dataset is larger than that in the first dataset, we clustered the dataset into different numbers of groups, such as 2, 3, 4 and 6. We then added a new variable into the experiments, which can be adjusted to achieve higher classification precision. When we first chose the best learning subsets from the results of SOM clustering based on the Wavelet feature with different levels, and then trained the neural network which is provided with the best learning subsets as input, the accuracy is shown in Table 3. In Table 3, *n* denotes the level of discrete wavelet transform and *c* denotes the number of clusters in the stage of the clustering analysis. The best performance with the decision rule of finding the best learning subsets in the second dataset is 77.71% when *n* = 5 and *c* = 2.

**Table 3:** Best learning subset: accuracy with different *n* and *c* in the second database

*n*\*c*	1	2	3	4	5	6	7	8	9
2	73.13%	71.88%	74.17%	71.88%	77.71%	73.75%	73.96%	73.33%	75.63%
3	67.50%	72.92%	66.67%	68.75%	71.25%	68.13%	70.21%	72.29%	72.71%
4	68.75%	67.29%	66.46%	68.13%	66.87%	67.08%	68.13%	68.75%	68.75%
6	63.75%	64.17%	61.67%	62.29%	64.38%	61.46%	62.50%	62.71%	64.38%

If we firstly train different neural networks based on the subsets from the SOM clustering based on a Wavelet feature with different levels, and subsequently combine the outputs of different neural networks by majority rule, the performance, shown in Table 4, is a little better than in Table 3. The highest accuracy reaches 78.96% when *n* = 9 and *c* = 2 (Table 4).

**Table 4:** Majority rule: accuracy with different *n* and *c* in the second database

*n*\*c*	1	2	3	4	5	6	7	8	9
2	71.88%	70.83%	73.13%	66.87%	68.75%	69.79%	75.00%	72.71%	78.96%
3	59.58%	66.25%	66.25%	64.58%	71.88%	67.71%	65.63%	67.50%	73.13%
4	67.08%	67.71%	63.33%	66.87%	63.33%	65.63%	65.83%	71.46%	72.08%
6	65.00%	65.63%	63.12%	60.83%	61.88%	63.33%	65.21%	64.38%	65.83%

## Discussion

In this section, we will discuss the influence with the parameters and data noise of this study.

### Parameters

In our experiment, it is found that the adjustment of parameters will affect the accuracy of classification. In the first experiment of PTB dataset, these two parameters m and α can be adjusted to achieve better classification performance in the electrocardiogram diagnostic system. Where, *m* denotes the number of bits in a word for the reduced binary pattern, and α denotes the scale factor of the interval of the reduced binary sequence. As the number of bits in a word used to simplify binary patterns increases, the performance generally becomes more and more positive. As the number of attributes in the feature vector is 2^*m*^, with the increase of *m*, more and more attributes in the feature vector, which means that we can extract more attribute information from the ECG signal for the diagnostic system. However, if the *m* value is too large, clustering and neural network will be very time-consuming and complex, resulting in poor performance, especially for small datasets. Thus, we need to determine the appropriate *m* and *α* ranges for the diagnostic system.

In the second experiment of TVGH dataset, there are two parameters, *n* and *c*, which are different from those in PTB dataset. In addition, *n* represents the level of DWT and *c* represents the number of clusters in the cluster analysis stage. In the first experiment, two parameters are used to extract RBP features of different scales. While the second experiment, *n* is used to extract wavelet features of different levels, and *c* is used to adjust the number of clusters in the cluster analysis stage. In addition, the number of clusters should not be too large, which may lead to the shortage of samples in the learning subset of the diagnostic system. Otherwise, we cannot choose the appropriate learning subset to train the network to get a better performance. Similar to the first experiment, we can add parameter *c* to the second experiment, although the process becomes complex and time-consuming due to these three parameters. This parameter may result in a better performance, which has certain value for further research.

### Data noise

Electrocardiography describes the electrical activity of the heart recorded by electrodes placed on the body surface. The change of voltage is caused by the action potential of cardiac myocytes. In the process of ECG recording, the signal may be interfered by low-frequency or high-frequency noise, so as to change the waveform of ECG signal. As mentioned above, the original data in the ECG dataset of PTB diagnosis is seriously affected by noise, while the signal quality in the TVGH dataset is significantly better than that in the PTB dataset. Noise has an impact on preprocessing, QRS detection, feature extraction, and even the final classification of ECG features. Therefore, this study needs to study the different noise characteristics of different ECG datasets in order to find the best feature processing method for each ECG dataset.

## Conclusions

With the development of computer analysis technology, the association between ECG dataset and medical record is likely to discover the association features that are not easy to be found before. In this research, the relationship between time series ECG and smoking was found by using clinical medical records, and two datasets were used to carry out relevant validation experiments.

The different ECG datasets may need different features of diagnostic system. In our experiment, we have determined the appropriate ECG features for the two ECG datasets, that is, PTB ECG dataset is suitable for RBP features and TVGC dataset is suitable for wavelet features. Through our designed neural network algorithm, we have achieved satisfactory results. The classification accuracy of the diagnostic system is more than 75–80%. which can judge whether the patients smoke at more than 75% level. Although the current method needs to adjust the parameters, such as adding parameter c in the experiment, we can study more automatic algorithm as the future work. Meanwhile with the popularization of the medical system [36], more ECG and medical record data should be collected in the future, than our proposed methods might be valuable to explore the relationship between more unknown diseases and medical records.

## Data Availability

The datasets used and analyzed during the current study are available from the first author upon reasonable requests. The dataset and material from Taichung Veterans General Hospital (TVGH) are publicly available via github with the link https://github.com/vghtcdata/vghtcdata.

## Abbreviations

ECG: Electrocardiogram
PTB: Physikalisch-Technische Bundesanstalt
RBP: Reduced Binary Pattern
CAD: Coronary Artery Disease
EMG: Electromyography
SOM: Self-Organising Map
BMU: Best-Matching Unit
DCT: Discrete Cosine Transform
DWT: Discrete Wavelet Transform
SA: Sino-Atrial
AV: Atrio-Ventricular
TVGH: Taichung Veterans General Hospital
